# Anatomy of the deltoid muscle trigger points

**DOI:** 10.1016/j.clinsp.2025.100795

**Published:** 2025-09-21

**Authors:** Leonardo Henrique Alves Rocha, Lucas Hara, Larissa Barbosa Lima, Ana Itezerote, Flávio Hojaij, Mauro Andrade, Alfredo Jacomo, Flavia Akamatsu

**Affiliations:** a Physiotherapist Department of Surgery, Laboratory of Medical Research-Division of Human Structural Topography, Faculdade de Medicina da Universidade de São Paulo (FMUSP), São Paulo, SP, Brazil; b Division of Human Structural Topography, Faculdade de Medicina da Universidade de São Paulo (FMUSP), São Paulo, SP, Brazil; c Department of Surgery, Laboratory of Medical Research-Division of Human Structural Topography, Faculdade de Medicina da Universidade de São Paulo (FMUSP), São Paulo, SP, Brazil

**Keywords:** Deltoid muscle, Trigger points, Myofascial, Nerve

## Abstract

**Background:**

Myofascial Pain Syndrome (MPS) is regarded as an extremely common clinical disorder, but is not fully understood. Shoulder pain has a lifetime prevalence of up to 66.7% in the general population, and pain due to Myofascial Trigger Points (MTPs) located in the deltoid muscle is fairly common. Analyses of the correlations between innervation patterns and trigger point locations have been successful in other muscular groups.

**Objective:**

The objective of this study was to provide a mathematical analysis of the entry points of the axillary nerve into the deltoid muscle and correlate them with the described areas of myofascial pain.

**Method:**

Ten cadavers were carefully dissected to measure a series of parameters in each deltoid muscle. The entry points of the axillary nerve into the deltoid muscle were placed in a cartesian graph created by dividing the deltoid muscle into 4-quadrants. Statistical analysis was carried out on the number of points in each quadrant.

**Results:**

Quadrant 1 had the largest number of axillary nerve entry sites, with a mean of 6.85 sites (63%), followed by quadrant 3 with 2.75 sites (25%), quadrant 2 with 1.15 sites (10%), and quadrant 4 with 0.25 sites (2%).

**Conclusion:**

In accordance with the clinical literature, the branches of the axillary nerve in the deltoid muscle corresponded to the described areas of the MTPs. Anatomical correlations between MTPs may be useful tools for gaining a better understanding of the physiopathology of these disorders and providing a basis for their treatment.

## Highlights

• First 3D chart of deltoid muscle nerve entrance sites, matching clinical trigger points.
• Anatomical evidence supports nerve entry sites as targets for precision therapies.
• Anatomy provides a scaffold for in vivo validation in clinical practice.

## Introduction

Myofascial pain syndrome is not fully understood. A recent study conducted by Shah et al., 2008^1^ revealed changes in the microenvironment of muscular fibers in individuals affected by myofascial syndrome through the introduction of a microcatheter and measurement of microenvironmental pH levels and inflammatory product concentrations. The initial presentation of myofascial pain has been described as a tense band, which may be asymptomatic. Psychological stress, muscular tension and other factors may transform the tense band into a trigger point.^2^

Diagnostic assessments of myofascial pain are not well established.^3^, ^4^, ^5^ The clinical characterization of myofascial pain is trigger points that contain sensorial components.^3^^,^^6^ Nociceptors are responsible for pain sensation as well as muscular spasms. The motor component of myofascial pain is caused by the excess of acetylcholine liberated from various motor plaques, which causes muscular contractions, the accumulation of muscle fibers, and the formation of tense bands that are pathognomonic of trigger points^7^

The prevalence of shoulder pain in the general population in developed countries ranges from 4% to 26% according to various studies^8^ and it is a fairly common condition that is reported in primary care.^9^

Studies have found a high prevalence of muscles containing active and latent MTPs with high local mechanical pain sensitivity and referred pain in patients with chronic nontraumatic shoulder pain.^10^, ^11^, ^12^

The majority of cases may be included in the spectrum of myofascial pain. Not all muscles are equally affected, and myofascial pain may mimic other important clinical conditions.^13^

Differentiation of myofascial pain from other conditions is important in the clinical context.^13^ A study in the Netherlands actively investigated shoulder pain in a number of healthy patients, finding a prevalence of 38% in patients with trigger points in the deltoid muscle.^10^ The most commonly affected muscle was the infraspinatus muscle.^10^ Shoulder complaints are usually identified as signs and symptoms in the deltoid, upper arm and scapular regions, including shoulder stiffness and reduced range of motion, often leading to limitations in daily activities.^14^ The deltoid muscle is located in the shoulder, with proximal fixation in the proximal part of the clavicle, acromion, and spine of the clavicle. Fibers converge inferiorly to a narrow and voluminous tendon, which inserts into the deltoid tubercle on the lateral side of the humerus diaphysis.^15^ The deltoid muscle is divided into three regions: anterior, medial and posterior. Electromyography studies have been able to discern between seven independent muscular groups.^16^

The deltoid muscle is supplied by the acromial and deltoid branches ramifications of the thoracoacromial artery, the anterior and posterior circumflex humeral arteries, and the subscapular arteries and the deltoid branch of the deep brachial artery. The deltoid muscle is innervated by the axillary nerve and the terminal ramification of the posterior fascicle^15^ and different parts of the muscle may act independently. The anterior fibers aid the major pectoralis muscles in directing the arm forward and in a medially rotated position.^15^

Posterior fibers act in the same direction as the latissimus, as well as that in which the teres minor muscle acts, driving the arm backward and in a laterally rotated position. The acromial portion of the muscle is multipennate and abducts the arm until the capsule to the supraspinous muscle is taut.^15^

Movements are executed in the plane of the scapular body, which constitutes the only possible plane of rotation in which the arm can elevate above the head. In abduction, the acromial fibers are strongly contracted. Movement outside of the plane of movement is prevented by the scapular and posterior fibers.^15^ The deltoid muscle works considerably during arm elevation in the scapular plane.^17^

In the first degrees of abduction, the force exerted by the deltoid muscle is upward, but the upward translocation of the humeral head is made impossible due to the synergic downward traction of the subscapular, infraspinatus, and teres minor muscles.^15^

The deltoid muscle facilitates the suprascapular muscle in resisting the downward dislocation of the arm under the force produced by body weight and executes a balance of the arm during ambulation.^15^

Trigger point referred pain is locally disseminated in the affected region (anterior, medium or posterior) of the muscle^18^ and there is still a lack of anatomical data relating pain perception to motor and sensory innervation.^19^

The locations of the trigger points correspond to the locations of the motor plaque zones and may be represented by a myofascial trigger point in the anterior superior third of the deltoid muscle, another point in the posterior inferior third of the deltoid muscle, and six points in the middle of the deltoid muscle.^18^ The physiological mechanism of trigger points is not fully understood.^3^^,^^4^^,^^19^ Little anatomic data is available on these structures that constitute a significant obstacle to the obtention of a complete physiopathological explanation and the clinical management of trigger points and myofascial pain. The authors have not found studies describing trigger point location with respect to axillary nerve distribution in the deltoid muscle.

**Fig. 1:**
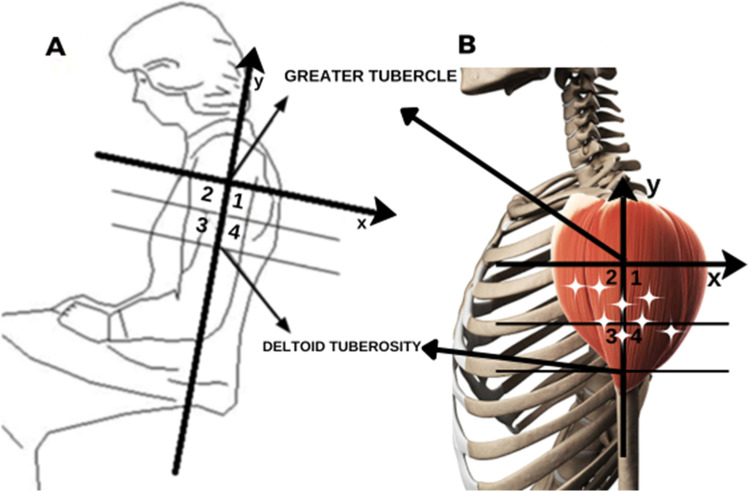
Scheme: (A) Quadrant divisions in the deltoid. (B) Quadrants with a representation of the deltoid muscle and stars that represent the trigger points by Simon’s scheme.^24^ Source: Own authorship, 2024.

Recent studies have demonstrated a relationship between the points of penetration of nerve branches in the muscle belly and the regions where MTPs have been clinically described by Travell and Simons, (1983)^20^ (Fig. 1B), Simons DG et al., (1999),^21^ and others.^22^, ^23^, ^24^, ^25^ In the deltoid muscle, there are two relevant MTPs, one in the anterior and the other in the posterior region of the shoulder, which produce a reduced range of motion.^26^ Therapies for myofascial pain include acupuncture, dry needling, physical therapy, and other behavioral measures.^27^ Defining the anatomical basis of TPs could provide a rational basis and therefore improve treatment efficiency by locating the structures involved in pain and avoiding injuries to them. The anatomical basis of the myofascial trigger points was reviewed by Ziembicki,^28^ supporting the hypotheses of the correlation of the muscle entry points with the trigger point phenomenon.^28^ He also pointed out the reproducibility and confidence level and of the method, which demonstrated the topographical superposition of the MTP with nerve branching to the muscles.^22^ According to Ziembicki, nerve entry points are anatomical characteristics of the clinical presentation.^28^ The studied group published studies of other muscles (e.g., gluteus maximus, abductor hallucis, masseter and temporal muscles) and found a correlation between trigger points and the anatomical identification of nerve entry points.^22^, ^23^, ^24^, ^25^

The anatomic study of deltoid muscle innervation and its relations to trigger points may prove to be of great importance in gaining a better understanding of the physiopathology of myofascial syndrome and other diseases related to lesions in the rotator cuff and may offer anatomic evidence for clinical and surgical interventions.

## Methodology

### Ethical aspects

This study was approved by the Ethics Committee of the Medical School for the Analysis of Research Projects Protocol 225/16.

### Anatomical technique

A similar methodology was used by Akamatsu et al. (2017) to evaluate the location of the points of penetration of nerve branches in the muscle belly via anatomical dissection.^23^

The authors performed left and right deltoid muscle dissection in ten cadavers of both sexes (six females and four males) to gather data on the patterns of innervation in the deltoid muscle. The cadavers belonged to the Discipline of Human Structural Topography of the Department of Surgery of the University Medical School and were obtained from a body donation program. The cadavers were fixed through anatomic dissection with a phenolic acid 4% and formaldehyde 0.5% solution. The specimens did not have any signs of previous surgery or any other severe abnormality in the areas studied. Information on sex, ethnicity, height, weight and age was gathered through the records from the Discipline of Human Structural Topography.

**Fig. 2:**
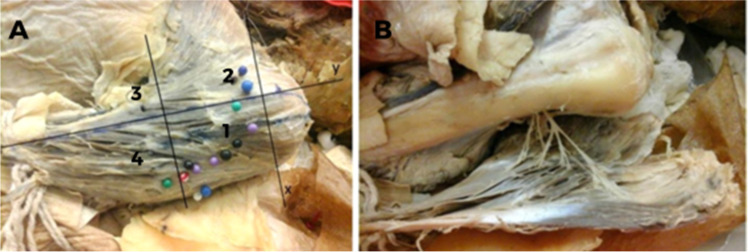
Left deltoid from a dissected cadaver. (A) Y-axis cross the humeral tubercle and the deltoid insertion point into the humerus. The tubercle is defined as the point (0, 0), and the numbers 1, 2, 3, and 4 represent the quadrants created in order to localize the insertions of the axillary nerve into the deltoid muscle. (B) Axillary nerve insertions into the deltoid muscle.

The specimens were positioned in decubitus dorsalis. The muscles were exposed through an extensive skin approach accompanying the anterior border of the deltoid muscle from the inferior margin of the acromion and clavicle until the deltoid tuberosity and included subcutaneous tissue, deep fascia and tissues overlying the deltoid muscle, which were deflected to expose the entire muscle belly. The muscle insertion and origin were sectioned, and the deltoid muscle was meticulously reflected to expose the vascular-nervous pedicles (Fig. 2A and 2B), which were marked with color pins to identify their entry points.

### Measurements of the deltoid muscle and delimitations of the quadrants

Morphometric measurements of the muscle dimensions and axillary nerve distributions were taken by using a Cartesian plane, with the axes based on bone references and the origin and insertion of the muscle. The distances between the acromion and the major tubercle of the humerus and between the humerus tubercle and the deltoid insertion were measured. A line perpendicular to the y-axis was traced halfway between the latter distance, creating another two quadrants for better localization of the coordinates (Fig. 1A and 2A). Therefore, four quadrants were formed in the negative part of the y axis, numbered from 1 to 4. Statistical analysis was performed using these four quadrants. The points of entry of the branches of the axillary nerve into the muscle were indicated with pins (Fig. 2A and 2B) and documented photographically using a Nikon D52 camera (Nikon Corporation; Tokyo, Japan). Penetration points were measured in relation to the median longitudinal and transverse axes by a simple division of values and classified according to the numbered area from 1 to 4.

### Statistical analysis

#### Sample calculation

The sample calculation was based on the results of the first 4 cadavers, 8 muscles evaluated as a pilot project, where the difference between quadrants 1 and 4 (Q1 and Q4, respectively) was 5.25 points on average with variability of approximately 4 points (SD = 3.9 points). Based on the results obtained in the pilot sample, with 90% power and 95% Confidence, the sample required to carry out the study is 20 muscles. As the present findings demonstrate a variation wider than one entry point, these sample reflects the expected results from a larger population. It was considered for the 2-sided test calculation for sex; muscles were described using summary measurements, and comparisons were made using Student’s *t*-tests. Mann-Whitney tests were used to compare the total number of points in each individual muscle according to sex, and the paired Wilcoxon test was used to compare the number of points between sides. The number of points related to each of the areas analyzed was described and compared between areas using generalized estimation equations with an interchangeable correlation matrix among sides and quadrants using a Poisson marginal distribution and identity binding function, followed by multiple Bonferroni comparisons to identify areas (1 to 4).

Analyses were performed using IBM-SPSS for Windows version 26.0 software. (IBM Corp., 2019. IBM SPSS Statistics for Windows, version 26.0. Armonk, NY: IBM Corp). The tests were performed with a significance level of 5% (p < 0.05).

## Results

### Specimens’ data

Ten specimens were dissected, totaling 20 deltoid muscles. Six cadavers were female, and four were male. The age of the cadavers varied from 56 to 91 years, with a mean age of 72.6 years. The height varied between 1.60 and 1.80 meters, with a mean height of 1.68 m. The weight varied between 50 kg and 80 kg, with a mean weight of 74 kg. Nine specimens were of White descent, and one was of Asian descent. Three specimens did not have height and weight data in the Discipline of Human Structural Topography records (Table 1).

**Table 1:** Description of the characteristics of the cadavers.

Variable	Description
**Sex, n (%)**	**(n = 10)**
Female	6 (60)
Male	4 (40)
**Right side**	**(n = 7)**
**Acromion-tubercle distance**	
Mean ± SD	4.1 ± 0,49
Median (min.; max.)	4.3 (3.2; 4.5)
**Tubercle-distal insertion distance**	
Mean ± SD	13.86 ± 0.73
Median (min.; max.)	13.8 (13; 15)
**Left side**	**(n = 9)**
**Acromion-tubercle distance**	
Mean ± SD	3.99 ± 0.32
Median (min.; max.)	4 (3.5; 4.5)
**Tubercle-distal insertion distance**	
Mean ± SD	14,13 ± 0,61
Median (min.; max.)	14 (13.5; 15.3)

### Dimensions of the deltoid muscle

Three measurements of the three right deltoid muscles and one of the left were not obtained. The measurement variation coefficient indicated homogeneity in the data (Table 2).

**Table 2:** Description of muscle measurements by sex and comparison results.

Variable	Sex		p	Variation coefficient
	Female	Male		
**Right side**	**(n = 5)**	**(n = 2)**		
**Acromion-tubercle distance (A)**			0.354	0.12
Mean ± SD	3.98 ± 0.55	4.4 ± 0.14		
Median (min.; max.)	3.9 (3.2; 4.5)	4.4 (4.3; 4.5)		
**Tubercle-distal insertion distance (C)**			**0.021**	0.03
Mean ± SD	13.5 ± 0.47	14.75 ± 0.35		
Median (min.; max.)	13.7 (13; 14)	14.8 (14.5; 15)		
**Left side**	**(n = 5)**	**(n = 4)**		
**Acromion-tubercle distance (B)**			0.384	0.08
Mean ± SD	3.9 ± 0.38	4.1 ± 0.22		
Median (min.; max.)	3.8 (3.5; 4.5)	4.1 (3.9; 4.4)		
**Tubercle-distal insertion distance (D)**			**0.026**	0.03
Mean ± SD	13.76 ± 0.23	14.6 ± 0.63		
Median (min.; max.)	13.7 (13.5; 14)	14.7 (13.8; 15.3)		

*t*-Student test.

Table 2 Measurements of the distance between the acromion and the major tubercle of the humerus (A-right; B-left) and between the humerus tubercle and the deltoid insertion (C-right; D-left), with mean, standard deviation and variation coefficient.

### Distribution of the axillary nerve (Figs. 3,4,5 and Table 3)

**Fig. 3:**
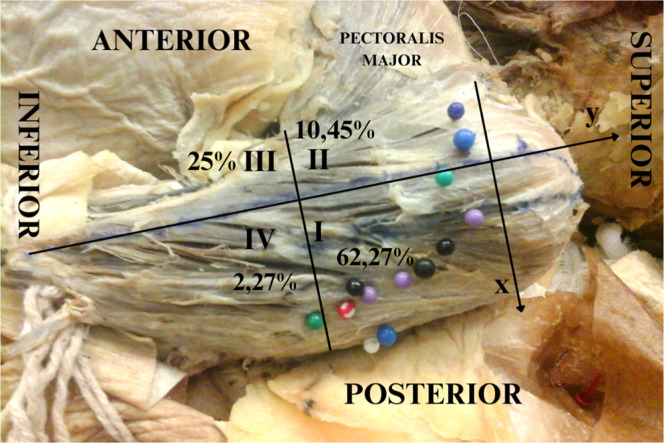
Left deltoid from a dissected cadaver. Y-axis cross the humeral tubercle and the deltoid insertion point into the humerus. The tubercle is defined as the point (0, 0), and the numbers I, II, III, and IV represent the quadrants created in order to localize the insertions of the axillary nerve into the deltoid muscle.

**Fig. 4:**
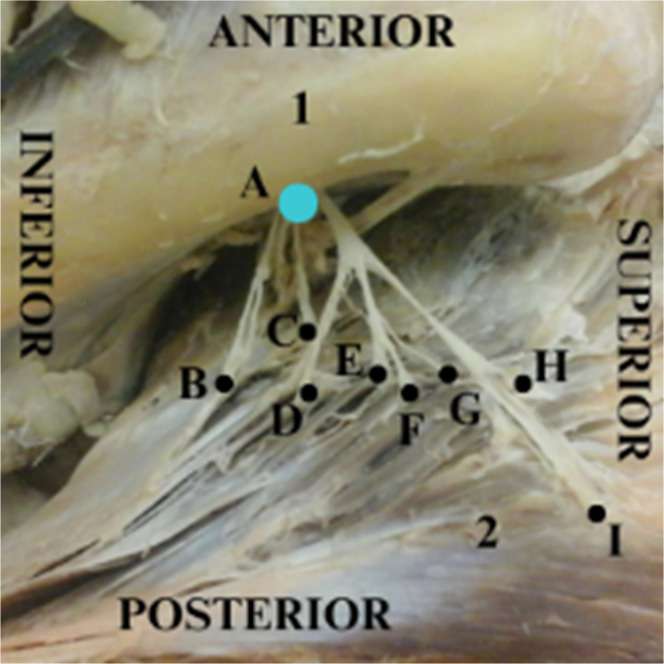
Left deltoid from a dissected cadaver. Axillary nerve insertions into the deltoid muscle. 1: Humerus; 2: Deltoid muscle folded A: Axillary nerve ‒ blue; B, C, D, E, F, G, H e I shows in black the branching distribution of the axillary nerve entering the deltoid muscle.

**Fig. 5:**
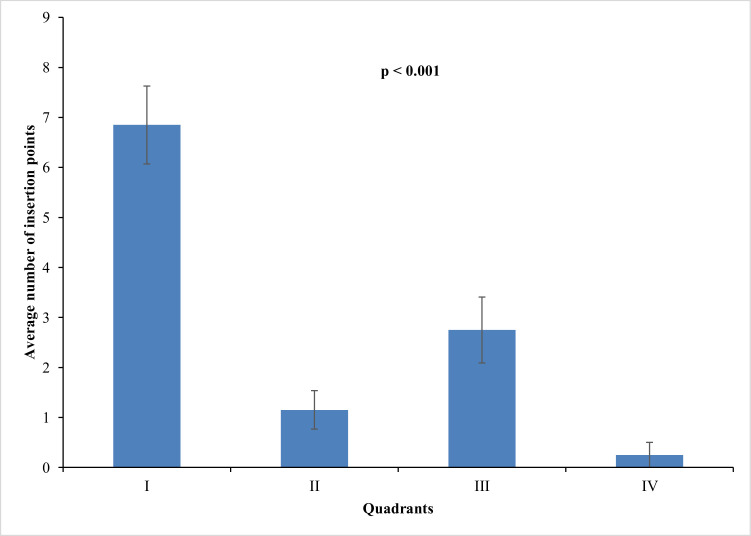
Number of axillary nerve insertion points into the deltoid muscle in regions 1, 2, 3, and 4. Source: Own authorship, 2025.

In a one-to-one comparison, the authors observed that region 1 has the most axillary nerve entry points compared with other regions, followed by regions 3, 2 and 4. The number of entry points (mean and standard deviations) is described in Table 3, Fig. 3‒4, and the dispersion graphs (Annexed) (Fig. 5).

**Table 3:** Description of the number of nerve entry points according to quadrants in the cadavers and results of the comparative test.

Quadrants	Points of entry		p
	Mean ± SD	Median (min.; max.)	
**1**	6.85 ± 3.48	7 (2; 16)	<0.001
**2**	1.15 ± 1.73	0 (0; 5)	
**3**	2.75 ± 2.94	2 (0; 10)	
**4**	0.25 ± 1.12	0 (0; 5)	

Note: GEE with Poisson distribution and identity link function, assuming an exchangeable correlation between the quadrants and sides of the muscles.

SD, Standard Deviation.

## Discussion

The deltoid muscle, a narrow and thick muscle, surrounds the glenohumeral articulation in all directions, except inferomedially, covering the round aspect of the shoulder.^21^ However, in the current literature, the authors have not found data about its dimensions. In the present study, the authors found that the mean dimensions of the deltoid muscle were homogeneous, with a small variability coefficient. The population was composed of adults (56 to 91 years old, with a mean age of 72.6 years), with a mean height of 1.68 m and a mean weight of 74 kg (ranging from 50 to 80 kg).

The distance from the major tubercle of the humerus to the deltoid tuberosity was larger in male specimens, probably due to the longer length of the humerus in this population. Additional studies are necessary to clarify these findings.

The data obtained in this study, through the observation and classification of axillary nerve entry sites into four quadrants in dissected specimens, showed variability between specimens in the number of axillary nerve insertions (the difference ranged from 1 to 16 insertion points), with no difference between the right and left sides.

Therefore, the authors may conclude that despite the large variability between specimens, the number of axillary nerve entry points in each specimen is maintained independent of laterality.

The distribution of axillary nerve entry points in the four quadrants the authors established in the deltoid muscle did not show a statistically significant difference (p < 0.05) between male and female specimens, except for quadrant 1. The authors found, however, a statistically significant difference in the number of axillary nerve entry sites in quadrant 1 between male and female specimens, which was not observed in quadrants 2, 3, or 4. In males, quadrant 1 had a statistically significantly larger number of axillary nerve entry sites. The authors suggest that this finding is related to the larger size of the muscle in males, but did not measure it.

The authors found the insertion points of the axillary nerve, predominantly in regions 1 and 3, reflected the location of trigger points in the medial region of the deltoid, as described by Simons et al., 2005.^18^ Dividing the muscle into quadrants possibly allows a more precise localization of the trigger points. The authors rarely found insertion points in the region superior to the major tubercle of the humerus, and this region is not discussed by Simons et al., 2005.^18^ The myofascial trigger point in the anterior region of the anterior third of the deltoid muscle was related to quadrant 2, where the authors found fewer insertion points of the axillary nerve, and the same observation was made for quadrant 4. The 6 myofascial trigger points located in the medial and posterior parts of the muscle were related to quadrants 1 and 3. In the present study, the authors found that the majority of points were located in these quadrants. Even quadrant 3 appeared to have fewer points, and the authors consider the insertion nerve points in this quadrant related to the medial deltoid.

Myofascial pain syndrome may cause pain, tension, sensation and limitations in movement for a certain muscle group.^10^ A study conducted by Alburquerque-Sendin F et al., 2013^11^ analyzed 27 patients with shoulder impingement syndrome and 20 healthy controls, with measurements of myofascial trigger points and pressure pain thresholds. Patients with shoulder impingement syndrome had more myofascial trigger points and a smaller pressure pain threshold, both on the affected side and on the healthy side, indicating a possible process of peripheral sensitization of pain.^11^ The central region of the deltoid muscle is multipennate, with 4 septa that initiate in the acromion and interconnect with 3 septa ascending from the deltoid tubercle.^15^ This structure may be related the finding of a larger number of axillary nerve entry sites in this region of the deltoid muscle, motor plaques of the angulated fibers in a multipennate region are more plentiful and distributed.^29^ The termination of the axon to the muscle fiber defines an area known as the endplate region. These endplates are usually located near the middle of the muscle fibers.^30^ The multipennate region of the deltoid muscle contains more muscle bellies than other regions, justifying the larger amount of nerve endings in these regions.

The position of axillary nerve entry sites in the deltoid muscle may aid in defining the position of trigger points in the muscle. The same approach has been successful in studies with the gluteus maximus muscles, abductor hallucis muscle and masseter muscle.^22^, ^23^, ^24^, ^25^

Possible therapies for myofascial pain include acupuncture, dry needling, physiotherapy, and other behavioral therapies.^27^

The efficiency of these methods has been researched. A review of the interventions by Sergienko and Kalichman, 2015^26^ suggested that these interventions are effective in reducing pain and improving the range of motion and function of the shoulder.^26^

The initial hypothesis that triggers points of the deltoid muscle corresponded to the branching pattern of the axillary nerve seems supported by the present findings, provided that they correspond to the clinical location of MTPs described by Simons and Travell.^20^

The anatomical basis of the myofascial trigger points was reviewed by Ziembicki T, (2023)^28^ supporting the hypotheses of the correlation of the muscle entry points with the trigger point phenomenon.^28^ He also pointed out the reproducibility and confidence level and of the present study’s method, which demonstrated the topographical superposition of the MTP with nerve branching to the muscles.^22^ According to Ziembicki, nerve entry points are anatomical characteristics of the clinical presentation.^28^

The relationship between axillary nerve entry sites and myofascial pain may also have important clinical and surgical therapeutic implications.^10^^,^^31^

There is an anatomical relation between the deltoid trigger points and the axillary nerve entry sites on the deltoid muscle belly. A better understanding of the nerve entry sites in the deltoid muscle and its topographic relationship with the locations of trigger points may have important clinical and surgical applications. Recently, Ultrasound (US)^32^ and Magnetic Resonance Imaging (MRI)^33^ studies have shown the existence of MTrPs and taut bands, which are localized areas of increased muscle stiffness that cause local constriction due to a taut band or are altered by inflammation or autonomic changes. The resulting ischaemia and hypoxia are thought to promote the release of chemicals that irritate and activate peripheral nociceptors, causing pain. This supports Simons' concept that MTrPs are caused by excessive acetylcholine release, resulting in aberrant contractions in a taut band.^20^^,^^34^

**Fig. 6:**
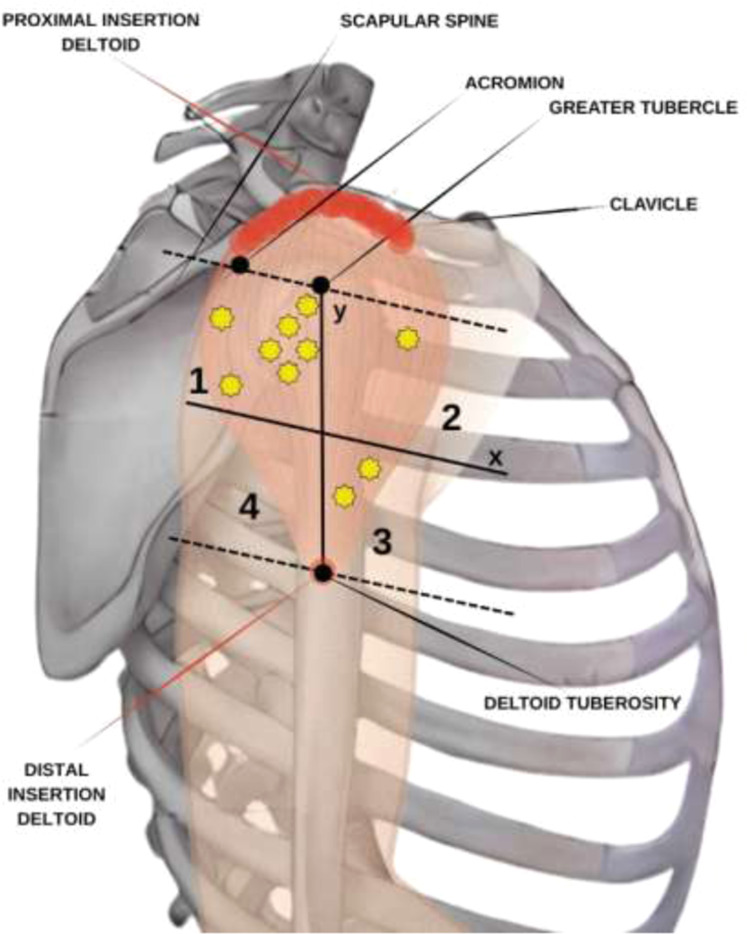
Diagram (‒) Quadrant divisions in the deltoid muscle. (–) Quadrants with a representation of the deltoid muscle and stars that represent the trigger points prevalence (%) found in the present work.

Research with US^32^ and MRI^33^ should be supported with the anatomical localization of the myofascial points on examining diagnostic test accuracy and reproducibility of data to establish the best performing methodologies. These exams can be guided by looking for the quadrants I and III with more nerve entry points on the deltoid muscle (Fig. 6).

Methods such as acupuncture, shockwave therapy, local anaesthetic injections, dry needling and various other therapies have been used to alleviate symptoms associated with abnormalities of the MTPs, with some authors reporting therapeutic benefits.^31^^,^^35^, ^36^, ^37^, ^38^, ^39^

This study provides an anatomy-based chart that may assist management of painful disorders in deltoid area. Injection of drugs such as botulinum toxin or anesthetics, as well as dry needling, may be made following anatomical guidance.

The parameters presented by this study may contribute toward a better understanding of the pathophysiological mechanisms of myofascial pain, which have not yet been fully elucidated.

## Limitation

The authors were unable to form groups in terms of age and race, as the cadavers they had access to were not available to choose from, but were cadavers from the Anatomy acquis. It is not possible to correlate myofascial trigger points in a living individual with the dissection using this method, as the authors cannot dissect in a living individual, which would lead to a clear understanding of the pathophysiology and diagnosis of myofascial disorders.

## Conclusion

The mathematical analysis of the entry points of the axillary nerve into the deltoid muscle was provided and correlated with the described areas of myofascial pain.

## Declaration of generative AI and AI-assisted technologies in the writing process

The authors declare that they didn’t use AI and AI-assisted technologies in the writing process.

## Funding

This research did not receive any specific grant from funding agencies in the public, commercial, or not-for-profit sectors.

## CRediT authorship contribution statement

**Leonardo Henrique Alves Rocha:** Investigation, Data curation, Visualization. **Lucas Hara:** Data curation, Methodology, Formal analysis. **Larissa Barbosa Lima:** Data curation, Methodology. **Ana Itezerote:** Data curation, Methodology. **Flávio Hojaij:** Writing – review & editing, Visualization. **Mauro Andrade:** Writing – original draft, Writing – review & editing, Visualization. **Alfredo Jacomo:** Supervision, Project administration. **Flavia Akamatsu:** Conceptualization, Methodology, Validation, Investigation, Resources, Data curation, Writing – original draft, Writing – review & editing.

## Declaration of competing interest

The authors declare they do not have any potential conflict of interest with regard to the investigation, authorship, and/or publication of this article.

## Appendix Supplementary materials

Image, application 1

Image, application 2
