# Supplementary material for: Anatomy of the deltoid muscle trigger points
Source: Clinics (Sao Paulo). 2025 Sep 21;80:100795. doi: 10.1016/j.clinsp.2025.100795 (PMC12489828; doi:10.1016/j.clinsp.2025.100795)
Supplement: Supplementary file 1 [file mmc1.docx]

**Anatomy of the deltoid muscle trigger points**

Leonardo Henrique Alves Rocha; Lucas Hara; Larissa Barbosa Lima; Ana Itezerote; Flávio Hojaij; Mauro Andrade; Alfredo Jacomo; Flavia Akamatsu

Division of Human Structural Topography, Laboratory of Medical Research 02 Department of Surgery Faculty of Medicine of the University of São Paulo (FMUSP), São Paulo, Brazil

Author contribution:

Leonardo Henrique Alves Rocha: Investigation, Data Curation, Visualization; Lucas Hara: Data Curation, Methodology, Formal analysis, Larissa Barbosa Lima: Data Curation, Methodology, Ana Maria Iterezote: Data Curation, Methodology, Flávio Hojaij: Writing - Review & Editing, Visualization; Mauro Andrade: Writing - Original Draft, Writing - Review & Editing, Visualization; Alfredo Luiz Jacomo: Supervision, Project administration; Flavia Emi Akamatsu: Conceptualization, Methodology, Validation, Investigation, Resources, Data Curation, Writing - Original Draft, Writing - Review & Editing
